# Improving social presence in online higher education: Using live virtual classroom to confront learning challenges during COVID-19 pandemic

**DOI:** 10.3389/fpsyg.2022.994403

**Published:** 2022-11-17

**Authors:** Aljawharah M. Aldosari, Saad M. Alramthi, Hala F. Eid

**Affiliations:** Faculty of Education, Bisha University, Bisha, Saudi Arabia

**Keywords:** digital learning, COVID-19, e-learning, MOOCs, learning analytics, sustainable online learning, virtual classroom

## Abstract

The COVID-19 pandemic has compelled practically all higher education institutions to adopt online education tools over the previous 2 years. Online education has a huge potential to supplement or take the place of in-person instruction. However, there are certain drawbacks of online learning, such as the absence of classroom environment interaction and the difficulty in keeping track of students’ engagement and participation. In this study, a live virtual classroom was developed to aid students in their learning activities. The effectiveness of these live video classes was reported from both students and instructors, as well as the variables promoting their implementation within higher education institutions. One of the more significant findings to emerge from this study is that the instructors found it convenient, as they could readily check course participants understanding by studying their live video lectures. The second major finding was that students felt satisfaction with online learning while asking questions without interfering with the instructor’s presentation. Moreover, peers could also provide them with more expertise. However, the teaching process became dynamic, requiring the educator to pay close attention. The course participants also experienced anxiety when they were in front of other people. Additionally, both the instructor and the students need to be highly self-sufficient in technology.

## Introduction

The United Nations Educational, Scientific and Cultural Organization, “UNESCO,” demonstrated that more than 1.5 billion students around the globe were forced to drop out of their educational institutions due to the emerging COVID-19 pandemic (United Nations Educational, Scientific and Cultural Organization (UNESCO), 2020). The pandemic has forced academic bodies around the world to discover new forms of learning and teaching, including online and digital education. New pedagogical methods were developed, more technological tools have been integrated in the teaching and learning process, and a focus on inclusion, resilience, and sustainability (Yousef and Khatiry, 2021). In fact, face-to-face instruction has unique properties, and no eLearning technologies can replace the physical presence of the instructor in a real classroom (El Miedany, 2019). However, due to COVID-19 isolation, learners and instructors might not be able to personally interact. Thus, online learning frequently acts as a complement, substitute, or even a replacement for in-person training. When it comes to accomplishing learning objectives, online learning can be just as effective as or even superior to face-to-face instruction (Anthony, 2019). Existing research recognizes the critical role played by instructional design, rather than only the use of new media or technology, which is mostly responsible for the comparable or higher learning results (Aldosari et al., 2022).

The development of the online tools’ technology for learning, particularly live virtual classroom, has made it possible to access the ideas and viewpoints among students’ network learning, creating chances for novel types of interaction and knowledge creation (Goldie, 2016). One of the most well-known network learning theories created for e-learning contexts is connectivism (Siemens, 2005). It is starting to gain acceptance among higher education instructors. On the other hand, the lack of in-person interactions in online learning environments poses a variety of difficulties for both instructors and students, including a lack of a collaborative learning environment, a loss of visual clues, and loneliness (Urrutia et al., 2015). Instructors and students must also adjust to the new settings, which are very different from traditional classrooms (Anthony, 2019). For instance, when talking to cameras rather than to actual students in classes, instructors and students could feel awkward or out of the ordinary. Similarly, rather than physically contacting the instructors, the students must watch recordings lectures of the instructors on their computer or smartphones. These difficulties and novel encounters will, in part, influence how the instructors deliver their lessons and how actively the students participate in online learning (Castelli and Sarvary, 2021).

Such approaches, however, have failed to address how live virtual classroom strategies are designed and implemented from the perspectives of instructors and students. Thus, there is an urgent need to address some issues of e-learning, including isolation and lack of social communication. In this paper, a group of students completing an online course at Bisha University was assisted by a live virtual classroom using Blackboard eLearning system. The instructor was physically apart from the students. This form was created as a synchronous classes run in real-time, interactive setting where the students and the instructors attending together from different locations utilizing a social networking application that enables both two-way video conferencing and real-time text chat. Therefore, the study’s objectives were to investigate how such a live virtual classroom supported by two-way video conferencing could be developed and deployed as well as to investigate the experiences and perspectives of the instructors and students for sustainable online education in higher education institutions in the post-COVID-19. The approach to empirical research adopted for this study seeks answer to the following questions:

1. What is the instructor’s reflection of the live virtual classroom’s design and implementation?
2. Do the live virtual classroom’s environments foster more social contact than other, more traditional distant learning models? Is this rise enough to give course participants a fruitful educational experience?
3. Which factors that influenced faculty members’ utilization of the synchronous virtual classroom and acceptance of it?

Qualitative data were collected from both students and instructor by using the questionnaires of the instructor’s feedback, and the students’ reflections. Moreover, learning analytics was used to analyze data about course participant and their contexts.

## Literature review

Digital education is an umbrella term for any type of learning represented using digital technology and includes students who take online courses. This form includes teachers who use digital tools, such as smart boards and portable devices, through synchronous and asynchronous learning tools (e.g., forums, shared discussion tools, email, chat, etc.,) with face-to-face interaction between students and instructors (Thomas, 2011). Thus, online learning is a form that is completely different from the traditional, in-person lesson within a department in an educational institution, depending on the time, the multiplicity of places, and perhaps the circumstances and capabilities (Rapanta et al., 2021). It is done by smart devices such as computers, smartphones, electronic boards and the like *via* the Internet, video conferences, audio–visual multimedia, radio, and television channels, through which a person, individually or a group of individuals, interacts with educational content determined by the professor or supervisors, whether it was a lesson, seminar, or training. However, the reported benefits such as improved learning outcomes do not occur directly within the online learning environment (Müller and Mildenberger, 2021). Instructional design plays a critical role in making an online learning environment more effective (Spatioti et al., 2022). Compared to face-to-face learning, the instructional design of digital learning has various challenges, some of which are to be elaborated in the following sections.

### Social isolation

There are numerous obvious advantages to learning in a classroom, including the development of a sense of belonging and high connectedness, the growth of positive relationships between students and the instructor and between students and peers, and the convenience of participating in learning activities (Eze et al., 2018). Due to the geographical separation, however, classroom climate is often scarce in an online learning setting. A key component of the classroom settings is social presence, which can be challenging to establish in an online learning setting (Wut and Xu, 2021). Social presence is the capacity of students to represent themselves socially and emotionally in an online context as “real” persons (Lowenthal, 2010). In an online setting, there may not be many opportunities for course participants and the instructor to interact. As a result, the atmosphere and social presence in the classroom may deteriorate, and the transactional gap between students and the teacher or among students may increase according to the principles of connectivism learning theory (Utecht and Keller, 2019). Connectivism was created by Siemens, who described it as a cutting-edge learning paradigm significantly influenced by using digital learning tools. Learners can distinguish between synchronous and asynchronous learning interactions by using a network, web, or Internet to (a) obtain updated learning material, (b) find reliable sources for their projects, and (c) knowing where to get information may be just as beneficial as the material itself (Abik et al., 2012). However, researchers attempted to evaluate the impact of utilizing technology, such as real-time video conferencing to establish a virtual classroom where the teacher and students may visually present themselves (Grassini et al., 2020). These experiments confirmed that the live virtual classroom could overcome the lack of social presence and classroom climate based on some requirements (Elmesalawy et al., 2021). The nature of eLearning media tools used can have a significant impact on social presence. Richer social cues can be provided by some medium, like video, than by others, like text (Binder, 2022). However, research also demonstrates that if participants engage in frequent communication and active interaction with one another, even text alone can establish social presence (Namaziandost and Nasri, 2019). It is undeniable that social presence is not solely influenced by the media used but is instead more based upon personal and social factors. However, the absence of nonverbal emotional cues in a communication system that only uses text severely restricts students’ capacity to detect the social presence of others (Heidari et al., 2020).

The manner in which communication media are used in eLearning systems can have a significant impact on social presence as well. For instance, asynchronous versus synchronous video usage would result in varying degrees of social presence. Asynchronously watching recorded videos would make students feel impersonal. In contrast, seeing a live video gives the course participants the impression that the teacher is speaking to them right away, which promotes a feeling of interpersonal connection (Lowenthal, 2022). Rich social presence improves students’ learning results. However, some studies show a significant relationship between social presence and learning success as well as social presence and learning satisfaction (Molinillo et al., 2018). Moreover, several lines of evidence suggest that improving social presence decreases the dropout rate of students from the online courses and improves student engagement (Sutherland et al., 2018). Social presence does not just mean an instructor’s physical presence among the students. According to additional research, students’ social presence among their peers is even more crucial for learning satisfaction than their instructor’s presence (Järvelä et al., 2016). This is because students are frequently more engaged in interacting with their peers than they are with the instructor (Kemp and Grieve, 2014). Thus, designing the online learning environment should place equal emphasis on establishing the social presence of students.

### Technological self-efficacy

Technological self-efficacy is the conviction that one can successfully use technology to fulfill a learning task (Pan, 2020). According to research, students’ motivation and contentment with the learning environment where technological tools are used are significantly influenced by their level of technological self-efficacy (Pan and Chen, 2021). Typically, utilizing a novel or unfamiliar technology instrument would enhance the students’ cognitive demands, anxiety levels, and likelihood of dropping out of a course. The adoption and use of technology by the instructor are also influenced by their level of technological self-efficacy. One of the main internal obstacles for instructors has been recognized as a lack of technological competency (Herro et al., 2021). When employing new technical tools to teach, instructors frequently experience nervousness (Larson et al., 2020). More recent attention has focused on the provision of training sessions that should not only concentrate on technical skills or elements, but also on how to use technology to effectively enhance teaching and learning activities (Heitink et al., 2016).

There are a number of large cross-sectional studies which suggest students’ participation is influenced by their level of technical self-efficacy. According to the case study conducted by Aguilera-Hermida et al. (2021) the students’ incapacity to use the technological tool was a major factor in the low engagement rate in an asynchronous video communication setting. In a different study, Lin and Wang (2021) found that students with lower technological self-efficacy had more trouble utilizing the tool and were less willing to participate.

### Track of students’ activities

Learning is a dynamic and interactive process. Monitoring student behavior and determining whether they are experiencing any challenges during the learning process is essential for good learning to occur (Weist et al., 2018). By keeping an eye on the learning process, the instructor can quickly alter the activities or content and enhance the course structure going forward. However, it can be challenging to keep track of students’ participation and engagement in an online learning environment (Ouyang and Chang, 2019). In the classroom context, the instructor may readily observe students and keep tracking on them using their body language, such as their gestures and facial expressions. However, in an online learning setting, it is challenging for the teacher to determine whether students are following along with the instructional content due to the inability to read students’ facial expressions (Wong and Li, 2020). In the synchronous online discussion, it is harder to keep track of students’ participation and engagement. In addition to giving instructional material, the teacher must pay close attention to and swiftly respond to any posts or requests made by students. Comparatively, in an asynchronous online discussion, the instructor typically has more time to evaluate students’ posts (Galikyan and Admiraal, 2019).

## Research method

**Figure 1:**
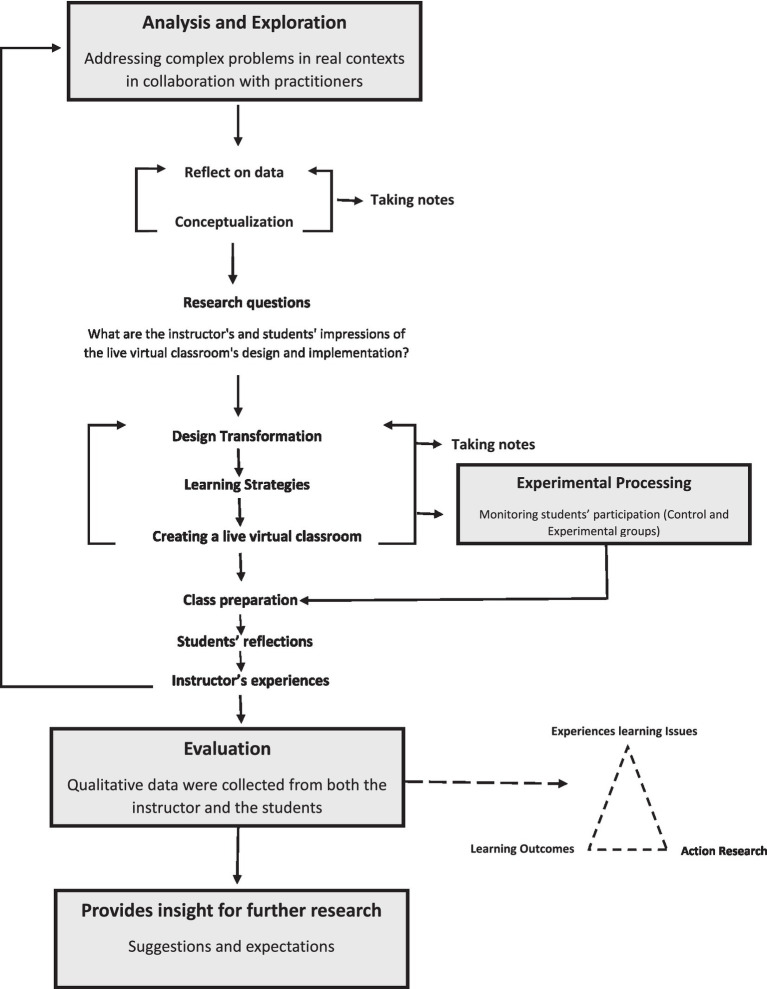
Action research method to improve social presence in virtual classroom.

This investigation takes the form of action research method. Action research can be defined as “an approach in which the action researcher and a client collaborate in the diagnosis of the problem and in the development of a solution based on the diagnosis” (Bell et al., 2022). One of the primary characteristics of action research is to investigate the partnership between the learning objectives and learning outcomes to address organizational issues as described in Figure 1. Based on this method, certain strategies were applied in the design and implementation processes to address the abovementioned challenges. In this section, the action research stages, i.e., analysis, exploration, strategies used, and course implementation process are to be described.

### Analysis

The live virtual classroom was designed for undergraduate students, Faculty of Education, University of Bisha, Kingdom of Saudi Arabia. The purpose of offering this course was to: (i) get the students to understand and recognize the importance of the pedagogical, social, and technical components of online learning environments and (ii) consider the design and evaluation of the components.

The virtual classroom was created as a real-time video conferencing environment where the instructor and students may engage concurrently in order to make up for the loss of social presence and classroom atmosphere. The instructor was available online during the meeting, frequently clarifying the course material and responding to students’ inquiries. Moreover, the teacher went over the material with the course participants and constantly quizzed them to make sure they understood. Additionally, in order to foster a feeling of community, the instructor could see the students on the screen in addition to the students seeing the live instructor video.

### Exploration

In this virtual classroom, the instructor could monitor students’ participation and understanding *via* the online status indicator, as their names would appear on the screen once individual students were online. The instructor could also get a sense of students’ understanding and engagement by glancing at their posted questions or comments on the discussion thread. Ideally, class discussion mainly consists of student participation; the teacher only facilitates and guides the students to keep the discussion going. Discussions are a great activity that adds vitality, excitement, participation, social interaction, reflection, and introspection to the dynamic of the classroom. The instructor could also use discussions as a formative assessment task—asking questions and brainstorming to check the current level of understanding. The constraint of the technological tool used in this course all members are allowed to see their status and show their activity, and they can exchange private messages with each other. *Via* watching the students’ live videos, the instructor could observe what they were doing and if they were following the discussion.

### Implementation

**Figure 2:**
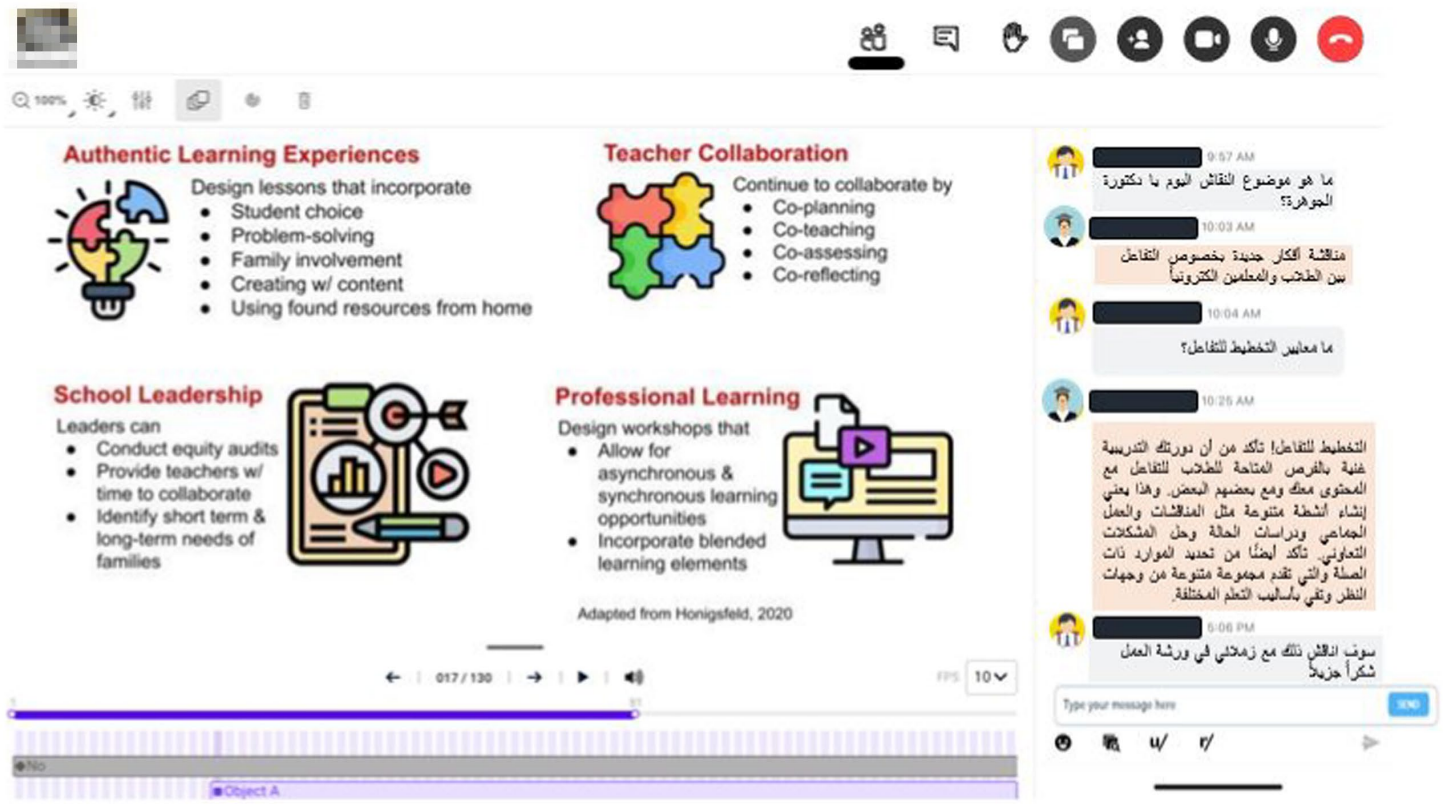
Virtual classroom at Bisha University using Blackboard learning management system (LMS).

Before the first lesson, the instructor held a try-out session with a few willing students. The teacher observed at how to distribute presentation slides and cast a live video. Moreover, the course instructor also looked at how to direct and coordinate students’ voices so that there would be minimal background noise and clear communication. The audio and video equipment’s functionality was also examined. On an as-needed basis, several extra training sessions were also conducted. For instance, the teacher is periodically required to provide students access to the materials’ files. Finally, the course instructor anticipated that the students would be able to view the shared screen, as well as see the highlighted text and remarks, as well as where the instructor had pointed his mouse on the digital whiteboard, as shown in Figure 2.

## Experimental procedures

The current experiment procedures were carried out by dividing the students into two groups (experimental–control), where the control group studied the traditional lecture system on Blackboard, while the experimental group studied the modified live classroom system as suggested by this study. Participants in this study were enrolled in the postgraduate faculty of education at Bisha University. Their ages ranged between 23 and 27 years old. The experimental group was 60 students, while the control group was 59 students. The procedures varied between activities managed before the beginning of the study lectures and activities managed during the lectures. This course aims to provide students with some cognitive and emotional aspects of learning related to teaching and its methods, which help them in teaching general education curricula.

### Before the class time

The lectures in both groups were about 120 min, including all activities, projects, and discussions, and the teaching continued for 12 weeks. For the experimental group, the instructor launched a new group video session before beginning a class, and a notification was instantly pushed to the chat window to let the students know. The instructor logged into the group video session and activated the computer camera to cast a live video. After joining the class, the students would watch the streamed video. Their names were added to the participant list automatically, allowing the teacher and other students to see who had attended the virtual session. On the other hand, the control group only had the possibility to follow some recorded lectures and presentations with discussion opportunities through asynchronous interaction tools.

### During the class time

In the experimental group, instructor and other students were able to join the live recordings, and students were asked to cast them to the virtual classroom. On occasion, the instructor would pose a question and the visible course participants would respond. The presentations were conducted in English, while questions were allowed in both Arabic and English. When a student’s name was called, they signed themselves up for the waiting list. The individual at the top of the waiting list took control of the microphone once the instructor released it and spoke into it in front of the class. The instructor could take over the microphone at any time if necessary. Only the instructor’s voice was broadcast to the students during a presentation. The student on the top of the waiting list started talking when he/she saw the microphone was released and stopped talking when the instructor took over the microphone. Very often the student started with ‘can you hear me?’ The instructor had to confirm by taking over microphone temporarily and then releasing it quickly for the student to continue.

## Results and evaluation

Since this was the first time the course had been offered in such a live virtual classroom setting during COVID-19, a formative course review was carried out to see what issues or challenges the students had encountered and how the course’s design and delivery may be further improved in the future. The following research question served as the basis for the course evaluation:

1. What is the instructor’s reflection of the live virtual classroom’s design and implementation?
2. Do the live virtual classroom’s environments foster more social contact than other, more traditional distant learning models? Is this rise enough to give course participants a fruitful educational experience?
3. Which factors that influenced faculty members’ utilization of the synchronous virtual classroom and acceptance of it?

### Instructor’s reflection

Conducting an online course in a live virtual classroom is a new experience for the instructor. As part of the course implementation process, the instructor, who was also a researcher, noted what was prepared before a session, observed, and noted what transpired during a lesson, and considered how the lesson may be improved. Throughout the data analysis process, each researcher personally reviewed the observation and reflection log numerous times. To show the instructor’s experiences and opinions of using the live virtual classroom, key issues were selected, debated, and common themes were condensed. The present results are significant in the following major respects.

#### Factors that influenced instructors’ utilization of virtual classroom

Sixteen items on the questionnaire measured the extent to which factors that influenced faculty members’ utilization of the synchronous virtual classroom and acceptance of it. Five-point Likert scale was used, where (1) Strongly Disagree, (2) Disagree, (3) Neutral, (4) Agree, and (5) Strongly Agree. The results obtained from the preliminary analysis are summarized in Table 1. Closer inspection of the table shows that online dialog with learners in real-time (*M* = 4.11) and offering a suite of collaborative tools (*M* = 4.12) had the highest mean among the collaboration factors. Promotes social presence among students (*M* = 4.17) and it allows for better student-instructor interaction (*M* = 4.28) were most highly rated among the social factors. Enhanced students learning outcomes (*M* = 4.26) and students having more time to reflect on what they learned (*M* = 4.72) had the highest average among feedback factors. The flexibility of technology (*M* = 3.94) and enriched student products and portfolios (*M* = 3.92) had the highest means when considering usability factors.

**Table 1:** Factors that influenced instructors’ utilization of virtual classroom (*N* = 12 instructors).

No	Category/factor	*M*	SD
*Collaboration*			
1	Online dialog with learners in real-time.	4.11	1.12
2	Offering a suite of collaborative tools, e.g., chat rooms and blogs.	4.12	1.16
3	Encourages shy students to interact with teachers better.	3.12	1.30
4	Students experience a stronger sense of community with their classmates.	3.15	1.17
*Social presence*			
5	Promotes social presence among students.	4.17	1.12
6	Allows for better student-instructor interaction.	4.28	0.98
7	Helping students with social integration despite the quarantine period.	3.86	1.36
8	Students have a direct connection with their peers.	3.50	0.95
*Feedback*			
9	Enhanced students learning outcomes.	4.26	1.13
10	Students had more time to reflect on what they learned.	4.72	1.18
11	Allows instructors to know which concepts and aspects of course design is beneficial to the students.	3.50	1.12
12	Students upload assignments digitally for peer review.	2.78	1.23
*Usability*			
13	The flexibility of technology.	3.94	0.91
14	Enriched student products and portfolios using learning analytics.	3.92	1.11
15	Perceived ease of use.	3.12	1.36
16	Enable instructors to share each other’s designs.	3.51	0.84

#### Adjustments of the instructor’s role

The instructor had to regularly examine the students to see if they were still following the discussion or if they had difficulties in understanding the content. Occasionally, during the instructor’s presentation, the students remained silent for a considerable amount of time without speaking. The teacher had to halt, elicit answers from the course participants, and ask questions. During the online classes, the instructor got incredibly concentrated. The instructor had to keep a careful eye on the chat window while presenting the material so that any questions or comments from the students could be noted and quickly handled. Multiple questions may occasionally be posted at once, and some may receive responses from peers. Before moving on with content delivery, the instructor had to swiftly evaluate whether specific questions or comments needed to be addressed.

### Students’ reflections

**Figure 3:**
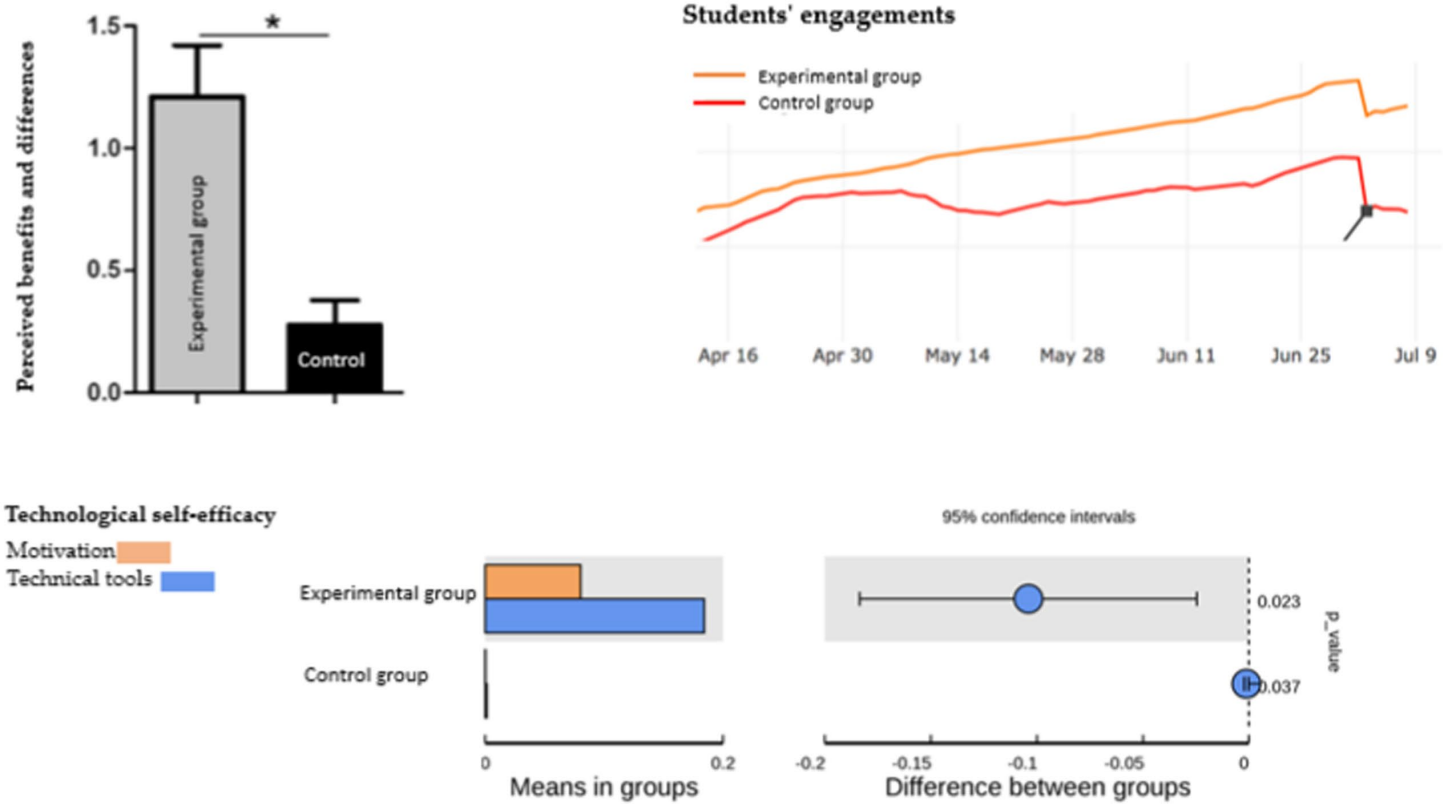
Statistical analysis among groups using learning analytics.

Do the live virtual classroom’s environments foster more social contact than other, more traditional distant learning models? Is this rise enough to give course participants a fruitful educational experience? In light of the results in Figure 3, it is clear that there is a noticeable superiority for the experimental group that was taught in the interactive video conferencing system compared to the control group that was taught in the traditional way. The majority of the students in the experimental group indicated that the interactive virtual classroom offered a convenient and welcoming setting where they could attend the course using their own computers at their preferred locations, such as the study room. The schedule for online learning was also flexible because the time of the class could be determined as needed. Therefore, most students could participate in each session. The course participants believed that they learned more from their peers. In a real classroom, students often exclusively pick up knowledge from the teacher. However, students received assistance and advice from other participating classmates in this online classroom using interactive learning tools. Additionally, they believed that the resources that their peers shared and the relationships with others were beneficial to them.

Regarding students’ engagement, the participants felt free to raise questions without interfering with the instructor’s speech. While watching the instructor’s lecture, they may quickly post questions to the chat window. The teacher or other students may also provide them with timely criticism. In contrast, students frequently hesitate in a face-to-face classroom to interrupt the instructor’s lecture and ask questions. Moreover, they thought that taking notes would be simple. Additionally, the chat window automatically captured the conversation and postings for later use.

## Discussion

In a real classroom, the students can easily see if the instructor is in the classroom and if the instructor is going to start a lesson. But in an online environment, these visual cues are missing. In this virtual classroom, the instructor informed the students of his presence by broadcasting a live video. Thus, we emphasize the necessity of starting the live lectures a few minutes before their time to adjust the settings and prepare for broadcasting at the correct time. This finding is consistent with that of Newton et al. (2014) who confirmed that video lecture should set up time of about 3 min before class. Sometimes, the instructor also played a test video on the screen before a lesson started for the students to check if their computers were ready for receiving videos with sound and let them know the instructor was ready. Moreover, teachers’ awareness of the substantial supports in enhancing students’ self-directed learning.

Compared to traditional classroom teaching, the students became active participants rather than passive information receivers. They gave comments to the content, asked questions, and encouraged their peers to share ideas. Also, the students became useful helpers and resource providers (Gharti, 2019). For instance, when the instructor was referring to a figure the presentation, some students quickly found that figure and posted it to the chat window for others to view. When a student met technical problems, other students provided helpful information promptly.

Coordinating voice communication in the virtual classroom was challenging but manageable. The instructor kept the student’s videos on the screen silent and cast his voice only to the students. When a student was invited to talk, his/her voice was broadcast to the class, but the instructor could take over the microphone at any time. As the students and the instructor were familiar with the tool and knew how to take and release the microphone, the voice communication process was rather smooth. This is one of the general criteria for designing educational courses recommended by studies (Mishra et al., 2020). Moreover, there are many factors that influence the use of live virtual classroom, including an instructor’s level of comfort with the technology, and offering a suite of collaborative tools, e.g., chat rooms and blogs. These results corroborate the findings of a great deal of the previous work in Siemens (2017) who emphasizes the role of social media in promoting distributed learning across networks, and this is what the current study has benefited from as it provided the opportunity for students to discuss through discussion rooms and blogs.

This study proved that offering a live virtual classroom through two-way live video conferencing helped establish a classroom environment and was advantageous to both the instructor and the students. From the students’ point of view, watching the instructor’s live video increased his or her presence in the classroom and created the impression that the teacher was always in touch with them. This conclusion supported the findings of Fatani’s (2020) study, which showed that synchronous learning generally improved student happiness, and teaching effectiveness was more dependent on teacher, cognitive, and social presence than on technology. Technology is still a crucial tool for instructors’ instructional activities, nevertheless. Therefore, it is best to develop a blended course to supplement future course delivery (Van Doorn and Van Doorn, 2014).

From the instructor’s perspective, by watching the students’ live videos, the instructor felt that he was talking to real people rather than to the computer screen. Also, the instructor could observe whether the students were following him or had difficulties in understanding the content However, this study also revealed that the students who were visually displaying on the screen appeared to be anxious. To a certain extent, this anxiety might hinder their participation and concentration (Hilliard et al., 2020). This finding echoes the importance that an online learning environment must have certain social affordances so that students feel safe and comfortable to participate (Zhang, 2021).

This study uncovered several challenges with online education. The process of teaching and learning, for instance, became more dynamic. Anytime, students are welcome to ask questions or make comments, and they expect the instructor to get back to them right away. It is possible that the learning process will not go exactly as intended. This result confirms that the social dynamic in online learning environments has changed (Dhawan, 2020), and instructors are urged to deliberately create a favorable social dynamic concurrently with the delivery of knowledge (Wendell et al., 2019).

In addition, appropriately moderating voice communication to keep a quiet but interactive learning environment was a challenge. Students often like talking to others in real-time (Broadbent and Lodge, 2021). However, this study discovered that when the sound from various people arrived at the same time, it would be excessively noisy or difficult to distinguish their conversation. Therefore, it is essential to appropriately manage voice communication. This research supports the idea that in a technology-mediated learning environment incorporating audio, instructors should maintain control over the use of the microphone (Prentiss, 2021).

### Technological self-efficacy

No glaring disparities were noticed when the online sessions were held at various locations. The Internet was generally rather fast, and there were no additional technical issues when the courses were held at a remote location. The instructor needs to feel comfortable using the technology. The instructor in this research has extensive experience using the technical instrument. However, familiarization sessions were still important and beneficial since they helped the instructor foresee what would happen and determine whether the intended function could be achieved. This finding is consistent with what Rim and Shin (2021) found that tutors valued the training as it part of education components, e.g., learning objectives, course flow, instructional design, feedback techniques, follow-up and evaluation procedures, debriefing structures, and human resources. This finding also confirmed that instructors must have technological knowledge in addition to the pedagogical and content knowledge as described in the TPACK (Technological, Pedagogical Content Knowledge) model (Lachner et al., 2021).

The students also need to be comfortable using the technology. In this study, the control group students were unable to finish the course in large part due to a lack of technological proficiency with the instrument. This result supported the hypothesis that students’ level of technical self-efficacy would influence their engagement in technologically mediated learning environments (Aldhahi et al., 2022).

### Implications

The study’s conclusions have ramifications for creating and putting into practice live virtual classrooms that use technology. The class size ought to be suitable. It might be difficult to keep track of students’ participation and engagement in real-time online learning environments. The instructor can use video conferencing to keep track of the students’ comprehension. However, a technological instrument, especially a free service, frequently does not support many live videos at once. As a result, each student in this session took turns introducing themselves graphically. If the class is large, it would not be possible to observe every student in a short amount of time.

Before a live online lesson, students need to be well-prepared, and scaffolding questions should be created and available beforehand. Students typically have to respond right away in a synchronous setting, as opposed to taking their time to reflect or research during an asynchronous online session. Without adequate preparation, it would be difficult for them to participate in the conversation and offer helpful suggestions. They could prepare for lessons more purposefully if scaffolding questions were provided beforehand. This would be especially helpful if the learning assignment was challenging, and the course participants felt overwhelmed by it (Zhang et al., 2021).

### Limitations and future studies

The generalizability of these results is subject to certain limitations. For instance, the instructor tried to avoid often changing the content on the shared screen due to the network speed restriction. The instructor may show a PPT presentation, and the students will see it together in the class video section. When the content on the shared screen was often moved between sources, it would take some time for the students’ screens to update (such as video and PPT). In order to avoid this delay, the instructor had to refrain from switching the screen frequently. Because of the nature of the department, the class size was rather modest. A small class was manageable for observation and communication, but a larger class would present more difficulties. Future studies would involve a larger number of participants to examine if instructors and participants have different experiences. In addition, the participants in the study were motivated and mature. The findings from this study cannot be generalized into other dissimilar contexts where participants are more junior or less motivated. Future studies would involve less mature students to confirm if the findings from this study are representative.

## Conclusion

With the spread of the Corona pandemic all over the world, schools and universities were closed as a precaution. E-learning *via* the Internet has become the ideal solution to confront this closure and to continue education. However, there are also limitations to online learning, such as the lack of connection in the classroom and the challenge in monitoring students’ participation and engagement. In this study, a live, virtual classroom was created to support student learning and to investigate teacher and student experiences with and impressions of the live virtual classroom’s conceptualization and implementation. The study used the quasi-experimental approach through two groups, one control and the other experimental. The sample amounted to 119 students, divided into the two groups, in addition to 12 teachers who participated in the final questionnaire to determine the factors that lead to an increase in the effectiveness of the use of electronic classes. The results showed a remarkable superiority of the students in the experimental group, and the social presence and psychological support for them were enhanced. Moreover, from the instructors’ point of view, the online dialog with learners in real-time and offering a suite of collaborative tools had the highest mean among the collaboration factors. Furthermore, promoting social presence among students and allowing students for better student-instructor interaction were most highly rated among the social factors. Enhanced students learning outcomes and students’ reflection on what they learned had the highest average among feedback factors. In addition, the flexibility of technology and enriched student products and portfolios had the highest means when considering usability factors.

## Data availability statement

The raw data supporting the conclusions of this article will be made available by the authors, without undue reservation.

## Author contributions

AA: conceptualization. HE and SA: methodology. AA, HE, and SA: validation and writing—original draft preparation. AA and SA: formal analysis, investigation, resources, data curation, visualization, supervision, project administration, funding acquisition, and writing—review and editing. All authors contributed to the article and approved the submitted version.

## Funding

This research was funded by Institutional Funding Initiative for Supported Scientific Research in KSA, University of Bisha.

## Conflict of interest

The authors declare that the research was conducted in the absence of any commercial or financial relationships that could be construed as a potential conflict of interest.

## Publisher’s note

All claims expressed in this article are solely those of the authors and do not necessarily represent those of their affiliated organizations, or those of the publisher, the editors and the reviewers. Any product that may be evaluated in this article, or claim that may be made by its manufacturer, is not guaranteed or endorsed by the publisher.
